# Anticancer Plants: A Review of the Active Phytochemicals, Applications in Animal Models, and Regulatory Aspects

**DOI:** 10.3390/biom10010047

**Published:** 2019-12-27

**Authors:** Tariq Khan, Muhammad Ali, Ajmal Khan, Parveen Nisar, Sohail Ahmad Jan, Shakeeb Afridi, Zabta Khan Shinwari

**Affiliations:** 1 Department of Biotechnology, University of Malakand, Chakdara 18800, Pakistan; 2 Department of Biotechnology, Quaid-i-Azam University, Islamabad 45320, Pakistan; parveennisar@yahoo.com (P.N.); shakeebafridi@outlook.com (S.A.); shinwari2008@gmail.com (Z.K.S.); 3 Department of Zoology, University of Buner, Sowari 17290, Pakistan; ajmal.sci@gmail.com; 4 Department of Biotechnology, Hazara University, Mansehra 21120, Pakistan; sjan.parc@gmail.com; 5 National Council for Tibb, Islamabad, Pakistan

**Keywords:** cancer, apoptosis, herbs, cell lines, in vivo

## Abstract

The rising burden of cancer worldwide calls for an alternative treatment solution. Herbal medicine provides a very feasible alternative to western medicine against cancer. This article reviews the selected plant species with active phytochemicals, the animal models used for these studies, and their regulatory aspects. This study is based on a meticulous literature review conducted through the search of relevant keywords in databases, Web of Science, Scopus, PubMed, and Google Scholar. Twenty plants were selected based on defined selection criteria for their potent anticancer compounds. The detailed analysis of the research studies revealed that plants play an indispensable role in fighting different cancers such as breast, stomach, oral, colon, lung, hepatic, cervical, and blood cancer cell lines. The in vitro studies showed cancer cell inhibition through DNA damage and activation of apoptosis-inducing enzymes by the secondary metabolites in the plant extracts. Studies that reported in vivo activities of these plants showed remarkable results in the inhibition of cancer in animal models. Further studies should be performed on exploring more plants, their active compounds, and the mechanism of anticancer actions for use as standard herbal medicine.

## 1. Introduction

The burden of cancer rose to 18.1 million new cases and 9.6 million deaths in 2018. With 36 different types, cancer mainly affects men in the form of colorectal, liver, lung, prostate, and stomach cancer and women in the form of breast, cervix, colorectal, lung, and thyroid cancer [1]. Treating cancer has become a whole new area of research. There are conventional as well as very modern techniques applied against cancers. A variety of techniques i.e., chemotherapy, radiation therapy, or surgery are used for treating cancer. However, all of them have some disadvantages [2]. The use of conventional chemicals bears side effects and toxicities [3]. But as the problem persists, new approaches are needed for the control of diseases, especially, because of the failure of conventional chemotherapeutic approaches. Therefore, there is a need for new strategies for the prevention and cure of cancer to control the death rate because of this disease.

Herbal medicine has become a very safe, non-toxic, and easily available source of cancer-treating compounds. Herbs are believed to neutralize the effects of diseases in a body because of various characteristics they possess [4]. For instance, among the many anticancer medicinal plants, *Phaleria macrocarpa* (local name: *Mahkota dewa*) and *Fagonia indica* (local name: *Dhamasa*) have been used traditionally for the anticancer properties of their active ingredients [5,6]. Metabolites extracted from the plant material are used to induce apoptosis in cancer cells. Gallic acid as the active component was purified from the fruit extract of *P. macrocarpa* and has demonstrated a role in the induction of apoptosis in lung cancer, leukemia, and colon adenocarcinoma cell lines [7,8]. It is a polyhydroxy phenolic compound and a natural antioxidant that can be obtained from a variety of natural products i.e., grapes, strawberries, bananas, green tea, and vegetables [9]. It also plays a critical role in preventing malignancy transformation and the development of cancer [10]. Similarly, other compounds such as vinca alkaloids, podophyllotoxin, and camptothecin obtained from various plants are used for the treatment of cancer.

With the advancement in the industrial sector and industrial medicine, the use of herbs was forgotten for a long period of time [11]. Hurdles regarding natural compounds are reduced because of the advent of new techniques and interest has been developed in the use of such natural ingredients in the pharmaceutical industry [12,13]. It has been estimated by the world health organization that 80% of the world is using traditional treatment methods [14]. Understanding of the effects or actions of herbs on various targets comes with the help of modern biomolecular science which recognizes some important properties i.e., anticancer, anti-inflammatory, and anti-virus. With the increasing understanding of the effects of such herbal medicine, their effects against different types of cancers have also been identified. For instance, hepatocellular carcinomas (HCC) are considered as the fifth most common malignancy in the world with increasing incidence [15,16]. Many studies have been performed on the treatment and prevention of using herbal medicine against HCC in which it is shown that all phases of HCC such as initiation, promotion, and progression could be affected by components of herbs [17,18].

However, as far as herbal compounds are considered as drugs, it is erroneously believed that they have no issues in terms of safety and side effects. There are hundreds of species of plants that are toxic to health. In the same way, there are many compounds in otherwise friendly plants that cause cytotoxicity. Based upon testing it has been proved that even anticancer plants result in cytotoxic effects [19].

Herbs are regulated under the “dietary supplement health and education act” as a dietary supplement in the United States of America. This review highlights the mechanism of some very important anticancer plants, the research related to their mechanism of action, their active ingredients, and the guidelines in place for their regulations.

## 2. Sources and Methodology

The most relevant literature was retrieved through a meticulous search on the electronic databases, Web of Science, Scopus, PubMed, and Google Scholar. The keywords and phrases used during the search were “Medicinal plants,” “Anticancer activity,” “Anticancer herbs,” “Anticancer plants,” “Mechanism of action,” “Animal models,” “in vitro activity,” and “in vivo activity.” The number of relevant articles finalized after extraction and analysis through the combination of the above keywords/phrases and the inclusion criteria was 200. The inclusion was based on two sets of criteria. According to the first set, i.e., “general criteria,” articles selected for this manuscript had (i) reported the traditional anticancer activity of plants and their parts, (ii) reported the anticancer role of extract or pure compounds from plants.

The second set of criteria was used for selecting specific anticancer plants whose phytochemicals are discussed in detail. For this purpose, twenty plants were selected for which recent articles were available that (a) studied in vitro and in vivo anticancer activities of herbal products, (b) reported the anticancer/antitumor activity of active compounds from the plants, and (c) assessed the in vivo anticancer activity of the herbal anticancer products.

All the data were extracted in a table and the mechanisms of action were explained in respective subheadings and demonstrated through different figures.

## 3. Selected Plants and Their Anticancer Activity

Research so far has tested the anticancer activity of a plethora of plants and plant-based compounds. Some of these plants and their compounds prove to be very effective against one or more types of cancers. Based on their activities, the following plants are selected for the in vitro and in vivo anticancer activities of their compounds. The rest of the important plants shortlisted for their activities are presented in Table 1 along with their activities.

### 3.1. Artemisia annua

**Figure 1:**
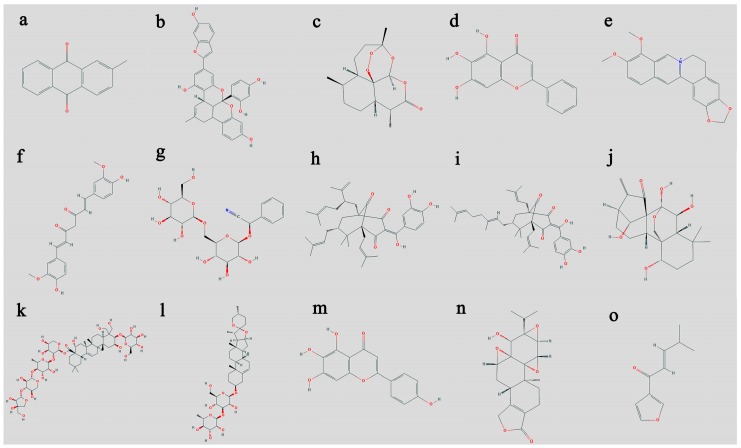
Structural representation of important anticancer secondary metabolites from plants. The structures are adapted from NCBI cited as National Center for Biotechnology Information. PubChem Database. (**a**) 2-Methylanthraquinone, Compound identification number (CID) = 6773; (**b**) albanol A, CID = 44567218; (**c**) artemisinin, CID = 68827; (**d**) baicalein, CID = 5281605; (**e**) berberine, CID = 2353; (**f**) curcumin, CID = 969516; (**g**) D-amygdalin, CID = 656516; (**h**) garcinol, CID = 5281560; (**i**) oblongifolin A CID = 53364454; (**j**) oridonin, CID = 5321010; (**k**) platycodin D, CID = 162859; (**l**) polyphyllin C, CID = 44429637; (**m**) scutellarein, CID = 5281697, and (**n**) triptolide, CID = 107985. (**o**) isoegomaketone, CID = 5318556; https://pubchem.ncbi.nlm.nih.gov/compound/ (accessed on 18 July 2019).

The genus Artemisia, widespread in Europe, Asia, North America, and South Africa has approximately 400 species worldwide [20]. Plants of the genus were used for centuries in classical medicine [21]. *Artemisia annua* is an annual short-day plant that belongs to family Asteraceae, having a brownish rigid stem. *A. annua* is known as sweet wormwood (Chinese: qīnghāo) and “dona” in the Urdu language in India and Pakistan [8]. *A. annua* was used by old Chinese for the preparation of anti-malarial drugs known as artemisinin (Figure 1). Having a unique ability of environmental adaptation it consistently resists insects and pathogens [22].

*A. annua* also synthesize scopoletin and 1,8-cineole compounds. Similarly, semi-synthetic derivatives of artemisinin are also generated such as arteether, artemether, and artesunate. Artesunate has been studied to be a very effective anticancer compound. Efferth [23] studied the effect of artesunate on 55 different cancer cell lines including leukemia, melanoma, lung cancer, colon cancer, renal cancer, ovarian cancer, and tumors of the central nervous system. They suggested that artesunate was most effective against leukemia and colon cancers. Furthermore, it was observed through these studies that the artesunate was more active than the drugs used for such cancers.

The stem and leaves *A. annua* were subject to extraction with the help of 80% ethanol and water. Several quantitative phenolic compounds from *A. annua* were identified using high-performance liquid chromatography (HPLC). The extracts were tested against HeLa and AGS cell lines. The cell growth inhibition activity of stem extracts was lower compared to leaf extracts. The ethanolic extracts of leaves lead to growth inhibitions (57.24% and 67.07%) in HeLa and AGS cells, respectively at a concentration of 500 mg/mL. HPLC analysis showed that the amount of phenolic acids was lower in stem extract than in leaves extract of *A. annua*. It was concluded from the data that the antioxidant and anticancer capacity was the result of phenolic compounds as well as unidentified compounds within *A. annua* [24].

### 3.2. Coptis chinensis

*Coptis chinensis*, the Chinese goldthread, is a herb used as a traditional medicine in China thus officially enlisted in the Chinese pharmacopeia [25]. It is widely known for its traditional use against various diseases like diarrhea, dysentery, acute febrile, and supportive infections. The organic extract of *C. chinensis* possesses anti-inflammatory and anti-oxidant properties [26,27]. *C. chinensis* extract has wide use in the treatment of cholera, dysentery, diabetes, blood and lung cancer because of its strong antibacterial activity [28]. *Coptis* genus contains the most important and active components, such as an alkaloid i.e., berberine (Figure 1). Berberines alkaloids are used frequently as criteria in the quality control of *Rhizoma coptidis* (Huang Lian) products and lead to the apoptosis of human leukemia HL-60 cells by down regulating nucleophosmin/B23 and telomerase activity. 

### 3.3. Curcuma longa

*Curcuma longa* (Turmeric) belongs to the ginger family Zingiberaceae. It is a rhizomatous herbaceous perennial plant [29]. It is naturally found in Southeast Asia and the Indian subcontinent. These plants are annually collected for their rhizomes and are then propagated from some of those rhizomes [30]. *C. longa* possesses a broad range of pharmacological activities including anti- HIC (human immunodeficiency virus), anti-inflammatory, antioxidant effects, nematocidal and anti-bacterial activities. 

Curcumin, the main component of *C. longa*, plays an important role in the therapeutic activities of *C. longa* [31]. Curcumin shows anticancer and anti-inflammatory activities as reported by many different studies. Cyclooxygenase (COX)-2 plays a vital role in the formation of colon cancer. In a study conducted by Goel et al. [32], the HT-29 colon cancer cells of humans were treated with different concentrations of curcumin to study the effect of curcumin on the expression of COX-2. The cell growth of HT-29 cells was inhibited by curcumin in a concentration- and time-dependent manner. Curcumin affected COX-2 by inhibiting its mRNA and protein expression, but no such inhibitory effect was found against COX-1. From this data, it can be suggested that the in vitro growth of HT-29 cells is significantly affected by a non-toxic concentration of curcumin. Curcumin may thus play an important role in the prevention of colon cancer. Furthermore, the anticancer effects of curcumin on human breast cancer cell lines (MCF-7) were assessed through lactate dehydrogenase and 3-(4,5-dimethyl-2-thiazolyl)-2, 5-diphenyl-2H-tetrazolium bromide assays to assess cytotoxicity and cell viability, respectively. The results showed that curcumin induced cytotoxicity and inhibited cells in a time- and concentration-dependent manner. This was observed through increased caspase 3/9 activity and induction of apoptosis. The results also indicated that curcumin downregulated miR-21 the expression of miR-21 in MCF-7 cells by upregulating the PTEN/Akt signaling pathway [33].

### 3.4. Fagonia indica

*Fagonia indica*, locally known as “dhamasa” is a flowering plant and belongs to the family of caltrop, Zygophyllaceae [34]. Members of Fagonia genus are known for their use as traditional medicine and are found effective in the treatment of many skin problems [35]. Traditionally, it was also used as a medicine for curing cancer as well as ailments resulting from poisons [5]. Amino acids and proteins [36], flavonoids [37], alkaloids [38], saponins [39], and terpenoid [40] are the phytochemicals found in the Fagonia species. *F. indica* is found to have liver protective [41] and antioxidant properties as well [42]. 

**Figure 2:**
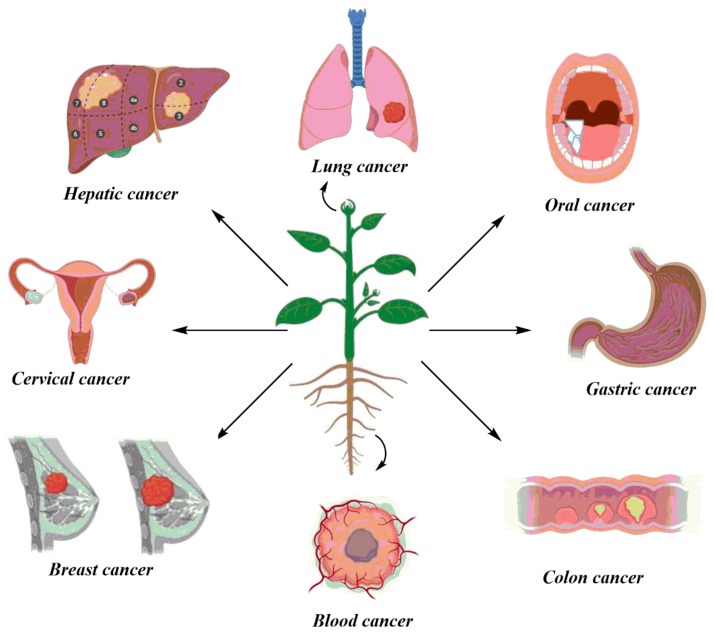
Illustration of activity of plants against several types of cancers. The icons were taken from Biorender illustrator and constructed through ChemBiodraw v14.0.

The aqueous extracts of *F. indica* have been found very effective against different types of cancer specifically breast cancers. For instance, Waheed et al. [43] performed bioactivity-guided fractionation to isolate the active and potent fraction of the *F. indica* extract. The activity was assessed against three cancer cell lines: MCF-7 estrogen-dependent breast cancer, MDA-MB-468 estrogen-independent breast cancer, and Caco-2 colon cancer cells (Figure 2). The results through different pieces of evidence such as the activity of pan-caspase inhibitor Z-VAD-fmk, caspase-3 cleavage, and DNA ladder assays suggested that apoptosis was stimulated in MDA-MB-468 and Caco-2 cells. Furthermore, a new steroidal saponin glycoside caused necrosis through cell lysis in MCF-7 cells. Similarly, Lam et al. [44] also demonstrated significant activity against breast cancer cells line MCF-7 through an aqueous extract of *F. indica*. 

### 3.5. Garcinia oblongifolia 

*Garcinia oblongifolia* (Lingnan Garcinia) belongs to the family of Clusiaceae and has a wide range of pharmaceutical activities. The important metabolites of the *G. oblongifolia* species; polyisoprenylated benzophenones and xanthones have anticancer, antioxidant, antifungal, apoptotic, and anti-pathogenic properties [45,46]. In vitro study showed that the bark of *G. oblongifolia* contains important secondary metabolites including oblongifolin A–G, oblongixanthones A–C along with other important compounds. These metabolites showed maximum apoptotic activities in HeLa-C3 cell lines and cytotoxic properties in the cervical cancer cells [47,48]. Li et al. [49] isolated about 40 different compounds from fruit, leaves, branches, and other parts of *G. oblongifolia*. They noted very high cytotoxic activities of these metabolites in the tested MCF-7 breast cancer cell line. However, they found the higher anti-cytotoxic activity of branch as compared to other plant parts. A small vacuole body formation was found at a low bark concentration of 0.250 g/mL. The vacuole size was increased at high concentrations of 500 g/mL and 1000 g/mL. The leaf part showed mild vacuole formation at a high concentration of 500 g/mL. Similarly, Feng, Huang, Gao, Xu and Luo [48] tested the pro-apoptotic activities of twenty different isolated compounds from *G. oblongifolia* in cervical cancer HeLa cells. Among all tested compounds the oblongifolins F and G, xanthone, nigrolineaxanthone T, and garcicowin B gave high pro-apoptotic properties at 10 μM concentration.

### 3.6. Garcinia indica

*Garcinia indica*, commonly known as kokum, is also an important medicinal plant that belongs to the Garcinia genus. The garcinol of *G. indica* shows positive activities in the experimental HT-29 and HCT-116 colon cancer cells along with normal immortalized intestinal cells (IEC-6 and INT-407). In another study, the fruit extract of *G. indica* was used for the isolation of garcinol. The garcinol at IC_50_ values (3.2–21.4 μM) for 72 h treatment shows strong inhibitory properties in all intestinal cells. The anticancer properties were higher in the cancer cells as compared to normal immortalized cells [50]. 

Similarly, Liao et al. [51] also observed a high tumor-inhibiting activity of *G. indica* in a human colorectal cancer cell line (HT-29). The garcinol at 10 μM concentration retarded the cell invasion activities several folds. The fruit extracts of *G. indica* has been shown very effective in the activation of caspase-3/CPP32 and the breakdown poly (ADP-ribose) polymerase (PARP) protein to inhibit leukemia in humans in the HL-60 cells [52]. These results indicated that garcinol (IC_50_ = 9.42 μM) shows strong growth inhibitory effects against human leukemia HL-60 cells.

### 3.7. Hedyotis diffusa 

*Hedyotis diffusa* (Chinese: sheshecao) is a member of the family Rubiaceae. It is spread over the northeast regions of Asia. *H. diffusa* has been commonly used to cure inflammatory diseases i.e., urethritis, bronchitis, and appendicitis [53,54]. 

Because of the recent advances in pharmacological practices, this herb received importance for having antitumor properties and showed effective results in treating cancers of the liver, colon, lungs, brain, and pancreas [55]. *H. diffusa* contains important bioactive derivatives of polysaccharides, triterpenes, and anthraquinones [56,57]. 

Methyl anthraquinonesare, one of the bioactive compounds in *H. diffusa*, is responsible for apoptosis of many cancers. It shows apoptosis and inhibitory effect on the MCF-7 cell line of breast cancer via activation of the caspase-4/Ca^2+^/calpain pathway when applied in a concentration of 18.62 µM for 24 h. It was observed that the S phase of the cell cycle and the percentage of the apoptotic cells were markedly increased when methyl anthraquinone was applied to MCF-7 cells [58]. Similarly, a concentrated extract of *H. diffusa* cause an inhibitory effect on the cervical cancer proliferation and induces apoptosis of Hela cells. Studies on the effect of *H. diffusa* ethanolic extracts on anti-colorectal cancer showed that these extracts cause an inhibitory effect on the Ct-26 cells by applying different concentrations (0.06 mg/mL, 0.08 mg/mL, 0.10 mg/mL and 0.12 mg/mL) with the rate of 35.46% to 71.84% [59]. 

### 3.8. Loranthus parasiticus and Scurrulus parasitica

*Loranthus parasiticus*, also known as Sang Ji Sheng (in Chinese), is a member of the Loranthaceae family and is widely distributed in the Southwestern regions of China. *L. parasiticus* is a semiparasitic plant, historically used as traditional folk medicine in China and Japan [60]. Since they are parasitic in nature, their biological activities including phytoconstituents are highly dependent on the host trees [61,62]. In fact, certain studies indicated the reverse effects of *L. parasiticus* on a host tree in decreasing sugar and chlorophyll content [63]. In vitro and in vivo studies have been conducted to evaluate the possible anticancer and antitumor potential of *L. parasiticus*. Cytotoxicity analysis of aqueous extracts of *L. parasiticus* has shown positive activity against ovarian cancer cell lines; SKOV3, CAOV3, and OVCAR-3 [64]. 

A comparative study conducted by Xiao et al. [65] on flavonoids extracted in 80% ethanol from *Scurrulus parasitica* harvested from different hosts showed a good anticancer potential on acute myeloid leukemia cell line HL-60. Flavonoids from *S. parasitica* on *N. indicum* induced apoptosis and inhibited cell proliferation with an IC_50_ value of 0.60 mg/L on HL-60 cells and arrested cell cycle at G_0_–G_1_ phase. *S. parasitica* parasitizing on *Morus alba* also showed an IC_50_ value of 2.49 mg/L. Similarly, Xiao et al. [66] isolated a polysaccharide in aqueous and ethanol extract from the leaves of *S. parasitica* and conducted proliferation inhibition assay on S180, K562, HL-60 cell lines. They observed inhibition in sarcoma S180 growth in mice with a 54% tumor inhibition rate Loranthus parasiticus on the optimal dose of 100 mg kg^−1^ d^−1^. *S. parasitica* downregulate expression of CyclinD1, Bcl-2, and Ki-67 protein, and upregulate the expression of Bax protein which helps in the inhibition of cancer cell line and apoptosis of cancer cell in vivo.

### 3.9. Morus alba

*M. alba*, commonly called white mulberry, is native to China, Japan, India and is cultivated throughout the world where silkworm is raised. Their leaves are the main source of food for silkworms. Extracts from *M. alba* are traditionally used to cure cough, edema, insomnia, bronchitis, asthma, nose bleeding, wound healing, eye infections, and diabetes [67]. *M. alba* contains many pharmaceutically important compounds like kuwanol, hydroxymoricin, moranoline, morusin, calystegin, albafuran, and albanol. The leaves of *M. alba* contain some active compounds such as quercetin, rutin, apigenin, 1-deoxynojirimycin [67].

A study by Chon et al. [68] on methanolic extract of *M. alba* leaves showed anti-proliferative effects on different human cell lines like pulmonary carcinoma (Calu-6), colon carcinoma (HCT-116) and breast adenocarcinoma (MCF-7). These results showed a strong link to the concentrations of the investigated extracts. Anti-proliferative activity in the methanolic extract of *M. alba* leaves was observed on the human cell line of gastric carcinoma (SNU-601) in a concentration of 1000 mg/mL.

In another study on albanol A, isolated from *M. alba* root extract, Kikuchi et al. [69] showed apoptosis-inducing, cytotoxic activity (IC_50_ = 1.7 µM) in HL-60 cell line. It induced topoisomerase II (IC_50_ = 22.8 µM), clearly reduced the levels of pro-caspases 3, 8, and 9. Furthermore, the Bax/Bcl-2 ratio also increased and induced HL-60 apoptotic cell death through stimulation of the death receptor. The results of a study conducted on *M. alba* leaves in different extracts on human cell line hepatoma (HepG2) showed that methanolic leaf extract also showed inhibition (IC_50_ = 33.1 μg/mL) of HepG2 cell. It was concluded that *M. alba* leaves extract contains different phenolic compounds in different solvents which showed an anti-proliferative effect on the HepG2 cell line through the arrest of the cell cycle in G2/M phase. This was achieved with p27^Kip1^ protein expression, activated caspases to induced cell apoptosis and inhibited topoisomerase IIα activity [70].

Furthermore, lectin was isolated from the leaves of *M. alba* which showed anti-proliferative activity on the human breast cell line (MCF-7) at a concentration of 8.5 μg/mL. This compound also showed cell cycle arrest and cytotoxicity in a human colorectal cell line (HCT-15) with inhibiting concentration of 16 μg/mL. The mechanism of inhibition of cancer cell lines was linked to the induction of apoptosis through activation and release of caspase-3 [71].

Methanolic extracts of *M. alba* showed significant anti-proliferative activity on the HepG2 cell line. These extracts showed significant inhibition of cells reducing cleared viable cell count. The cell growth was inhibited by suppressing the activity of NF-kB gene expression and biochemical marker modulation [72].

Further, Qin et al. [73] isolated 15 compounds from the root bark of *M. alba* which contain six diels-alder adducts and nine prenylated flavanones. The study observed that two new compounds, soroceal B and sanggenol Q showed cytotoxic activity. One of the isolated compounds showed selective cytotoxic activity in cells (HL-60 and AGS) at inhibiting concentrations of 3.4 µM.

### 3.10. Paris polyphylla

*Paris polyphylla* (called “Love Apple”) belongs to family Liliaceae and contains 24 species throughout the world [74]. *P. polyphylla* is mostly used by Indian and Chinese traditional medicine system for having potential anticancer properties. *P. polyphylla* consists of important secondary metabolites such as polyphyllin D, formosanin C, β-ecdysterone, dioscin, daucosterol heptasaccharide, oligosaccharides, octasaccharide, protogracillin, trigofoenoside A, yunnanosides G-J, padelaoside B, pinnatasterone, and other saponins [75]. Steroidal saponins are the main active components because of its structural diversity and bio-activities such as antitumor, immune-stimulator, analgesic, and hemostatic properties [76,77,78,79,80,81].

Aqueous and ethanol extracts of *P. polyphylla* showed potential antitumor activity against human liver carcinoma (HepG2 and SMMC-7721) cell line, human gastric (BGC-823) cell line, human colon adenocarcinoma (LoVo and SW-116) cell line, and human esophagus adenocarcinoma (CaEs-17) cell lines. Ethanolic extract showed a strong inhibitory effect with IC_50_ values ranging from 10 µg/mL to 30 µg/mL [82]. Extract of *P. polyphylla* also showed an antitumor effect in esophageal cancer ECA109 cells by increasing the connexin26 mRNA and protein expression. Studies reported that *P. polyphylla* extracts increased the *Bad* genes expression and decreased the expression of *Bcl-2* genes, inhibiting the growth of ECA109 cells by proliferation and inducing cell apoptosis [83].

### 3.11. Perilla frutescens

*Perilla frutescens*, commonly called perilla or Korean perilla or Beefsteak plant, is widely distributed in Vietnam, China, Japan, and most Asian regions belong to the Labiatae family [84,85]. Economically, one of the most significant crops, cultivation of *P. frutescens* in China and some other Asian countries is more than 2000 years old [86,87]. Stem, seed, and leaf parts of *P. frutescens* have been used to treat poisoning, cold, bloating, and headache [74,88]). Multiple in vivo and in vitro studies have been conducted to evaluate the anticancer and antitumor potential of *P. frutescens*. Leaf extract of *P. frutescens* showed the highest anticancer activity in HepG2 cells through cell proliferation inhibition and upregulation of apoptosis-related gene expression [89]. Other studies revealed that ethanolic leaf extract of *P. frutescens* promoted apoptotic induction and tumorigenesis through death-mediated receptors and scavenging the reactive oxygen species (ROS) [90,91]

Similarly, Lin, Kuo, Wang, Cheng, Huang and Chen [89] used leaf extract of *P. frutescens* to evaluate the proliferation and apoptosis in HepG2 cells. Anti-proliferation activity was observed in HepG2 cells treated with *P. frutescens* leaf extract at a concentration of 105 μg/mL. The study reported that significant apoptosis was observed through flow cytometry. Furthermore, microarray results showed apoptosis-related gene expression in a time-dependent manner. The activity of *P. frutescens* leaf extract was compared to the activity of rosmarinic acid which showed less effective results in apoptosis-related gene expression and apoptosis induction in HepG2 cells. 

The essential oil component “isoegomaketone” isolated from *P. frutescens* also induced apoptosis in human colon cancer (DLD1) cells. A study by Cho et al. [92] reported the inhibition of cell growth by isoegomaketone when treated for over 24 h, cleaved caspase-3, 8, and 9 in a time-dependent and dose-dependent manner. Isoegomaketone treatment triggered PARP cleavage, translocation of the protein Bax, cleaved Bid protein and the release of cytochrome *c* to the cytoplasm from mitochondria, induced apoptosis and translocation of apoptosis-inducing factor from mitochondria into the nucleus. It was suggested that isoegomaketone from *P. frutescens* induced apoptosis in DLD1 cells via both caspase-dependent and caspase-independent pathways. 

### 3.12. Platycodon grandiflorus

*Platycodon grandifloras*, commonly known as balloon flower, or Chinese bellflower, belongs to the family Campanulaceae, which is distributed through Northeast Asia. The rhizomes of *P. grandiflorus* are very effective and are used as a traditional medicine in China, North Korea, and Japan for treatment of different diseases like cough, sore throat, phlegm, and other ailments [93]. *P. grandiflorus* contains many biologically active compounds which include saponins, flavonoids, anthocyanins, phenolics, and polysaccharide. These compounds have significant immune-stimulatory [94], anti-inflammatory [95], hepatoprotective [96], and antitumor activities. The antitumor activity of *P. grandifloras* was shown in a dose-dependent manner by reducing PKC enhancement of matrix metallopeptidases (MmP-9 and MmP-2), which caused the death of HT-80 cells [97]. Yu and Kim [98] isolated platycodin D from the root of *P. grandiflorus* and treated MCF-7 cells with a concentration of 5–100 µM which reduce cell viability and proliferation in a dose-dependent and time-dependent manner as compared with controlled cells. Induction of anticancer activity was observed because of caspase 8 and 9 activation and PARP cleavage. In addition, platycodin D upregulate cellular levels of protein Bax and Bcl-2 and downregulate the activation of caspase-9. Further, it also induces proteolytic activation of Bid (a protein of the proapoptotic Bcl-2 family).

Platycodin D is a triterpene saponin isolated from the roots of *P. grandiflorus* shows cytotoxic effects on the human leukemia cells. It inhibited telomerase activity and showed a cytotoxic effect in a dose-dependent manner with a concentration of 10–20 µM. This was shown to be achieved through downregulating the expression of human telomerase reverse transcriptase (hTERT). The results of the study by Kim et al. [99] showed that suppression of telomerase activity and cytotoxic effect on leukemia cells by platycodin D was through post-translational and transcriptional inhibition of hTERT. Platycodin D induced apoptosis and cell death of human leukemia (U937) cell by inducing the production of ROS through *Egr-1* gene activation and as a result, decreased in mitochondrial membrane potential, activating caspase-3 and PARP cleavage [100]. 

The extract of *P. grandiflorus* also showed antitumor effects on ovarian (SKOV3) cancer cells. Results showed that induced apoptosis occurs through the downregulation of Bcl-2 expression, upregulation of Bax expression, activation of caspase (3, 8, 9), and mitochondrial cytochrome *c* released to the cytosol [101].

### 3.13. Prunus armeniaca

*Prunus armeniaca* (Armenian plum) belongs to an important plant family Rosacea. Various parts of the plant are used as the major source of some important antioxidant substances and are commonly used against cancer and some other cardiovascular diseases [102]. The fruit part of *P. armeniaca* contains various important secondary metabolites like β-carotene, flavonoids, organic acids, thiamine, minerals, and oils [103]. The seeds of *P. armeniaca* contains plenty of cyanogenic glycosides, used against different types of cancers [104]. Amygdalin is one of the important glycosides of *P. armeniaca*, used for the treatment of prostate cancer [105]. Gomaa [106] reported the antioxidant and anticancer activities of *P. armeniaca* at different combinations of methanolic and ethanolic extracts with water. The study concluded that kernels of *P. armeniaca* have better antioxidant capacity compared to almonds that belong to the same genus. The study showed the highest antioxidant activity (67%), total lycopene content (4.70 mg/mL), and total phenolic content (3.290 mg/g of dry extract) in *P. armeniaca* kernels. Furthermore, *P. armeniaca* kernels were also found active in the inhibition of certain cancers when tested on the colon (HCT-116), human breast (MCF-7), and HepG2 cell lines in different concentrations and combinations. The highest cytotoxicity was observed from methanolic extract (IC_50_ = 10.1 μg) against HepG2 cells. According to Madrau et al. [107], the fruit part contains a large amount of phenolic compound that shows strong antioxidant and immune-stimulant properties. Similarly, the study conducted by Liu et al. [108] found low anti-scavenging activity against DPPH of dry seed extract of apricot. Vardi et al. [109] reported the strong protective activity of apricot against intestinal oxidative damage in the rat model experiment. 

### 3.14. Rabdosiae rubescens

*Rabdosiae rubescens* (Chinese: Dong Ling Cao) is a Chinese medicinal herb that belongs to the family Lamiaceae. It possesses multiple biological activities like antibacterial, anti-inflammatory, anti-parasitic, and anticancer [110]. *R. rubescens* contain important chemical compounds including monoterpenes, sesquiterpene, diterpene, and terpenoids. Oridonin, a tetracyclic terpenoid, is the main active compound in *R. rubescens* [111]. Oridonin gained its attention because of the remarkable properties of growth inhibition and the induction of apoptosis in cancer cells. In vitro and in vivo studies showed the induction of apoptosis in a variety of cancer cells by oridonin as in hepatocellular carcinoma, breast, gastric, skin, colorectal, gallbladder, and pancreatic cancers [112].

Bao et al. [113] isolated oridonin from *R. rubescens* which showed a potent anticancer potential in gallbladder both in vitro and in vivo. SGC996 and NOZ cells treated with oridonin results in inhibition of colony formation and growth of tumor cells in S-phase and induced apoptosis. Wang, et al. [114] showed the molecular mechanism of oridonin as an anticancer compound in HepG2 cancer cells. Oridonin in a concentration of 41.77 μM inhibited the growth of HepG2 cells while in a concentration of 44 μM, it induced G2/cell cycle arrest and apoptosis when applied for 24 h. The expression of nine different proteins was observed through proteomic analysis. Eight out of the nine proteins are already reported to be involved in anticancer activity. Oridonin up-regulate STRAP, Hsp70.1, Sti1, TCTP, and PPase, downregulate hnRNP-E1 which are significantly involved in the G2/M cell cycle arrest and apoptosis. The upregulation of HP1 beta and GlyRS by oridonin are responsible for the inhibitory effects on tyrosine kinase and telomerase. 

Wang et al. [115] also reported that oridonin from *R. rubescens* is a potent cytotoxic agent that inhibited the growth and migration of highly metastatic human breast cancer cell lines (MCF-7 and MDA-MB-231). Oridonin significantly inhibited the growth of human breast cancer cells in a dose- and time-dependent manner, by arresting the cell cycle in the G2/M phase and accumulated cells to SUB-G1 phase. Results from the study also showed that oridonin triggered apoptosis by reducing Bcl-2/Bax ratio, NF-κB (p65), caspase-8, phospho-mTOR, IKKβ, IKKα and increasing the expression level of PPARγ, PARP, and Fas cleaving in a time-dependent manner. Oridonin significantly suppressed MDA-MB-231 cell invasion and migration through decreasing MmPs expression and regulation of integrin β1/FAK pathway and inhibited the growth and apoptosis via DNA damage and activated the extrinsic and intrinsic apoptotic pathway in breast cancer cells.

### 3.15. Scutellaria baicalensis 

*Scutellaria baicalensis* is one of the important medicinal plants species of family Lamiaceae. It is commonly known as Baikal skullcap or Chinese skullcap and is found in different regions of the world including East Asia, Europe, and the Russian Federation. Its root part is known as *Scutellariae radix* and used as traditional Chinese medicine for the treatment of hepatitis, respiratory, and gastrointestinal diseases [116]. The root parts have maximum flavonoid content having multiple pharmacological properties [117].

About 60 different flavonoids have been identified in *S. baicalensis* which showed maximum antioxidant activities [116,118]. The four flavones metabolites also showed antimutagenic properties [119]. Woźniak et al. [120] studied the antioxidant potentials of four flavones: baicalein, baicalin, wogonin, and their glucuronides compounds and wogonoside. These flavones have different antioxidant capacity depending on the chemical structure and mechanisms of activity. However, among these, baicalein showed maximum antioxidant activities while the wogonin provided high protection to the linoleic acid from oxidation but did not show any antioxidant activity [121]. 

The *S. baicalensis* extract is useful against a wide range of cancer cells like brain tumor cells [122], prostate cancer cells [123], and head and neck squamous cell carcinoma (HNSCC) cell lines [124]. The aqueous extracts of roots led to programmed cell death, and thus inhibit the growth and development of apoptosis. They suppressed the growth of lymphoma and myeloma cell lines via disturbing normal expression level of *Bcl* and *c-myc* genes, while increased the expression level of cyclin-dependent kinase inhibitor p27 (KIP1) [121]. Hongwei et al. [125] used three different concentrations (80, 120, and 160 μmol/L) of baicalin for 2 days against BGC-823 and MGC-803 gastric cancer cells. The results of 3-(4,5-dimethyl-2-thiazolyl)-2,5-diphenyl-2-H-tetrazolium bromide assay showed a lower rate at different doses. Flow cytometric analysis showed that baicalin induces apoptosis in a dose-dependent way. Also, it was found that baicalin increase the gene expression of caspase-3, caspase-9, and other B cell lymphoma (Bcl-2)-associated X protein but lowers the expression of the *Bcl-2* gene.

A recent study reported the angiogenic properties of baicalin by using chick embryo chorioallantoic membrane [126]. The aqueous extract of baicalin showed both inhibitory and angiogenic activities while the baicalein showed only anti-proliferative property. Quantitative PCR results showed the expression of 84 different angiogenesis-related genes at different doses of baicalin and baicalein. The low dose significantly increases angiogenic genes expression while the high concentration showed the opposite activity by decreasing angiogenesis and increasing the lethality. The low to high levels of baicalein decrease the level of gene expression of multiple angiogenic genes and decreased cell proliferation.

Sato et al. [127] studied the anticancer potential of the root extract of *S. baicalensis* by using human oral squamous cell carcinoma (OSCC) cell line. The findings of the study showed 100 µg/mL root extract retard the monolayer and anchorage-independent growth rate many times but do not affect the cell adhering ability. Downregulated expression of cyclin-dependent kinase 4 cyclin D1, G1 phase arrest, and PARP cleavage was observed with the application of the root extract of *S. baicalensis*.

### 3.16. Scutellaria barbata

*Scutellaria barbata*, the barbed skullcap is a key medicinal plant species of family Lamiaceae, used to treat inflammatory and cancer diseases [128]. It is rich in important secondary metabolites like alkaloids, flavones, steroids, and polysaccharides [129,130]. In vitro studies showed positive activities against a vast range of cancers i.e., colon cancer, lung cancer, hepatoma, and skin cancer [128]. 

The apigenin and luteolin isolated from *S. barbata* gave cytotoxic activity against both human breast cancer cell line MDA-MB-231 and non-transformed breast cell line (MCF10A) [131]. Similarly, scutellarein was found to possess strong anti-breast cancer activity demonstrated in MDA-MB-468 cell lines [132]. Scutellarein increased the concentration of mitochondrial superoxide and peroxide while decreasing the level of glycolysis, retarding the growth of cancer cells by lowering ATP synthesis. Other secondary metabolites of *S. barbata* showed cytotoxic properties by leading ROX and DNA damage [131]. The other important alkaloid of *S. barbata*, scutebarbatine A (SBT-A) also resulted in high antitumor and apoptosis activities in experimental A549 cells. 

### 3.17. Tripterygium wilfordii 

*Tripterygium wilfordii* of the family Celastraceae is also known as “Thunder God Vine,” and is native to Korea, China, and Japan. It is commonly used for the treatment of multiple diseases such as rheumatoid arthritis, systemic lupus erythematosus, nephritis, asthma, and cancers [133,134,135]. *T. wilfordii* produces important bioactive compound triptolide which is used as an immunosuppressive and anti-proliferative agent [134]. It has a five-membered unsaturated lactone ring and is used against different breast cancer cells by activation of pro-apoptotic compounds by modulating several signaling pathways [136]. 

In vitro studies showed anti-proliferative and pro-apoptotic activities against tumor cell lines [137,138,139]. He et al. [140] studied the in vitro properties of triptolide of *T. wilfordii* activity by using human umbilical vein endothelial cells (HUVECs). The IC_50_ value for HUVECs proliferation was 45 nM. The results of semi-quantitative RT-PCR and Western blot analysis in human umbilical vein endothelial cells showed that triptolide cause downregulation of proangiogenic Tie2 and VEGFR-2 expression after 24 h at 50 nM concentration. At high concentration (100 nM), the VEGFR-2 mRNA expression was completely blocked. But on the other hand, the knockdown of Tie2 decreases the inhibitory activities of triptolide on endothelial network formation. The overall results showed that anticancer properties of triptolide are directly correlated with the blockage of two endothelial receptor-mediated signaling pathways. Triptolide showed more antagonistic activities against the proliferation of HUVECs as compared to normal cells like skin keratinocytes HaCaT cells and other liver cells L-02 [141].

Celastrol is another important compound of *T. wilfordii*, having cytotoxic activity against a broad range of cancer cells [142]. It demonstrates its anticancer activities by blocking the NF-κB via targeting IκB kinase and TAK1-induced NF-κB activation [143,144]. Other studies showed that triptolide of *T. wilfordii* has anticancer properties in many model systems such as experiments have also demonstrated triptolide’s therapeutic efficacy in several model systems including neuroblastoma in nude mice model [145], other xenografts of human melanoma, breast cancer, bladder cancer, gastric carcinoma [146]. 

### 3.18. Tussilago farfara

*Tussilago farfara* (commonly called coltsfoot) is one of the important medicinal plants, grown in Europe and various regions of western and central Asia, commonly used against cancer. It possesses a high quantity of flavonoids and other phenolic compounds and some trace elements (Zn, Mg, and Se). The presence of these substances plays a key role in the anticancer activities of this plant. Maximum scavenging activity was recorded in water extract as compared to ethanol extract. It shows a 20.9% antioxidant activity. Further, this plant showed maximum antioxidant activity both using DPPH and yeast model [147]. The quercetin-glycosides isolated from the flower bud of *T. farfara* shows the highest antioxidant activity [148]. Lee et al. [149] reported the (TF)-induced cytotoxic and apoptotic activities of the flower part of *T. farfara* in human colon cancer cell line (HT-29) by using a methanolic extract. Fatykhova et al. [150] showed the genotoxic activity of *T. farfara* herb juice against known genotoxic compounds like nalidixic acid in SOS chromotest and furacilin in Rec assay. Their findings showed that dilution of the herb juice gave maximum antimutagenic properties in SOS chromotest as compared to furacilin in Rec assay.

Lee et al. [151] reported the activity of *T. farfara* as the TNF-related apoptosis-inducing ligand (TRAIL)-induced apoptosis via MKK7/JNK activation by inhibition of mitogen protein kinase-TOR signaling pathway regulator-like protein (MKK7-TIPRL) in human hepatocellular carcinoma cells. The *T. farfara* extract decreases 50% interaction between MKK7-TIPRL. HPLC data further verified the presence of many important phenolic compounds that decrease MKK7-TIPRL interaction and also examined the activation of MKK7/JNK.

### 3.19. Wedelia chinensis 

*Wedelia chinensis* (Chinese: Peng qi ju), indigenous to India, South-East Asia, and China, is one of the important anticancer plants belonging to family Asteraceae which is rich in many important secondary metabolites like phenol, flavonoids, and tannin [152]. 

The essential oils of *W. chinensis* give a positive effect on lung cancer during the in vitro study. The GC-MS analysis recorded the presence of two important compounds carvacrol and trans-caryophyllene. High anti-scavenging activities were found at different levels of dose. The study of B16F-10 melanoma metastatic cell line showed that the concentrations of some important antioxidant enzymes (including catalase, superoxide dismutase, and glutathione peroxidase) increased many folds in the treatment groups. Similarly, the amount of glutathione also increased while the concentrations of other compounds such as lipid peroxidation and nitric oxide were decreased. The histopathology studies further verified that these essential oils show negative effects on cancer development [153]. 

## 4. In Vivo Studies of Anticancer Herbal Medicine: An Overview

**Figure 3:**
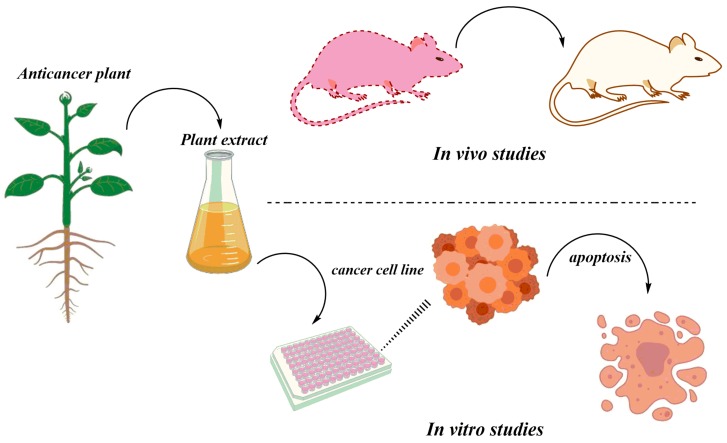
A depiction of general strategies applied for assaying extracts/phytochemicals from important medicinal plants for their anticancer activity both in vitro and in vivo.

The herbal medicines are tested both in vitro and in vivo. The anticancer activities of the various medicinal plants have been tested in vivo using different animal models (Figure 3). There are many studies available on in vivo experiments of the many different anticancer plants in mice models. For instance, dihydroartemisinin was reported to inhibit tumor tissue, increase the level of interferon-gamma (IFN-γ), and decrease interleukin 4 (IL-4) in tumor-bearing mice [154]. Similarly, artesunate, a derivative of artemisinin is also reported to be a promising drug against angiogenic Kaposi′s sarcoma [155], growth inhibition of A549 and H1299 lung tumors by 100 mg/kg dose [156], the suppression of human prostate cancer xenograft [157] and the inhibition of leukemia growth in mice [158]. 

Irradiation of C57BL/6 mice combined with a dose of 2 mg/kg twice a week was proved effective against lung carcinoma [159]. The effectiveness of berberine was enhanced when it was used in combination with other agents. Coptisine, another alkaloid of *Coptidis rhizoma* is proved to have anticancer effects when used in concentrations of 150 mg/kg against BALB/c nude mice by suppressing tumor growth and reducing cancer metastasis. The inhibition of the RAS-ERK pathway was suggested as the mechanism for this activity [160]. Another study was also performed on the nude mice on the HepG2 cells by applying the aqueous extract of *H. diffusa* which inhibits proliferation of cells in a dose-dependent manner, also delay S phase and arrest cells in G_0_/G_1_ phase [161].

Similarly, a high anticancer activity of SBT-A was found in transplanted tumor nude mice. Yang et al. [162] reported the anticancer activity of the polysaccharides isolated from *S. barbata* by 95-D Xenograft model. The results showed that polysaccharides give strong anti-proliferative activities against a 95-D cell line. It also lowered the expression of phospho-c-Met and other signaling elements like phospho-Erk and phospho-Akt. In vivo study also gave maximum antitumor activity by using a 95-D subcutaneous xenograft model. After one daily intraperitoneal injection for 3 weeks, the tumor growth was significantly decreased (47.72 % and 13.6%) at 100 and 200 mg/kg treatments. The ex vivo studies also showed that polysaccharides of *S. barbata* inhibit the phosphorylation of c-Met signaling pathway.

Furthermore, Li et al. [163] isolated a steroidal saponin from *P. polyphylla* which inhibited tumor growth in Lewis bearing-C57BL/6 mice and induced apoptosis in A549 cells. Results showed that steroidal saponin in concentration of 2.5, 5.0, and 7.5 mg/kg showed significant inhibition rate of 26.49 ± 17.30%, 40.32 ± 18.91%, and 54.94 ± 16.48%, remarkably increased thymus and sleep indices, decreased inflammatory cytokines (TNF-α, IL-8, and IL-10). This in turn inhibited the tumor growth in C57BL/6 mice by reduced volume and weight of tumor. Nuclear changes, DNA condensation, chromatin fragmentation, and apoptosis are induced in A549 cells with a concentration of 0.25, 0.50, and 0.75 mg/mL steroidal saponin. Tumor growth inhibited by steroidal saponin was associated with decreased ROS, inflammatory response, and induction of apoptosis.

Furthermore, Wanga et al. [164] reported the effect of isoegomaketone from *P. frutescens* on Huh-7 hepatoma cell carcinoma and tumor-xenograft nude mice. Results showed that isoegomaketone inhibited cells and decreased tumor weight and volume. Isoegomaketone in the concentration of 10 nM/L decreased pAkt without affecting Akt. Hepatoma cell carcinoma tumor growth was suppressed by isoegomaketone from *P. frutescens* through PI3K/Akt signaling pathway blocking. *R. coptidis* is also showed anticancer activity in rats as suggested by the inhibition of cyclooxygenase 2 activity. The number of aberrant crypt foci in the rat colon was decreased by 54% after the administration of *R. coptidis* extracts [165].

Manjamalai and Grace [166] reported the apoptosis along with lowering angiogenesis and lung metastasis activities of the essential oils of *W. chinensis* by using B16F-10 melanoma cell line in C57BL/6 mice. The mice were injected with B16F-10 melanoma cells through the tail vein and treated with different doses of essential oil. A 50-µg essential oil concentration showed maximum cytotoxic activities with 65.17% lethality within 24 h. The numbers of apoptotic cells increased many times in experimental samples as compared to the control group. They also recorded high levels of important proteins like p53 and caspase-3 in essential oil-treated samples compared to other non-treated samples. They recommended this plant for the treatment and control of cancer.

In vivo activities of oridonin from *R. rubescens* showed a potent anticancer potential in the gallbladder [113]. When injected intra-peritoneally with a concentration of 5, 10, 15 mg/kg for 3 weeks to athymic nude mice, oridonin significantly inhibited NOZ xenografts growth. Oridonin also inhibited NF-κB nuclear translocation, increased Bax/Bcl-2 ratio, activated caspase-3, caspase-9, and PARP-1 which showed that the mitochondrial pathway is concerned with apoptosis mediated by oridonin.

Studies have reported anticancer activities of two artemisinin dimer, dimer-hydrazone (dimer-Sal) and dimer-alcohol (dimer-OH) and one monomer dihydroartemisinin (DHA) compared to the control against MTLn3 breast tumors in rats. Results of the study reported that dimer-Sal, dimer-OH, and DHA significantly suppressed tumors in rats compared to the control group. It was also observed that the dimers were more potent as compared to the monomers [167]. 

It is also reported that artemisinin is responsible for preventing breast cancer in rats treated with a single oral dose (50 mg/kg) of 7,12-dimethalbenz anthracene (DMBA) which is known for rapidly inhibiting the multiple breast tumors. After the feeding DMBA with 0–2% artemisinin to the target group and plain food in powdered form to the control group, both groups of experimental rats were monitored for breast tumors for 40 weeks. Oral artemisinin significantly reduced the development of breast tumors (57%) as compared to control fed (96%). The research indicates that artemisinin might be a potent cancer chemoprevention agent, having lesser side effects [168]. Similarly, oral administration of curcumin to rats reduced the level of Gp A72 (glycoprotein) by 73% hence lowering paw inflammation [169].

Tanaka et al. [170] observed activities of fruit extracts of *G. indica* in the azoxymethane (AOM)-induced colonic aberrant crypt foci in male model rats (F344). They found lower proliferating cell nuclear antigen index and high concentrations of glutathione S-transferase and quinone reductase. They also observed the maximum chemo-preventive activities of garcinol. 

Besides mice and rats, there are many other animal models employed for studying anticancer activities. Zebrafish models are also employed for technical advantages including the ease of advanced genetic studies, expression of tumor in any organ and the striking resemblance to human malignancies [171]. Zhu et al. [172] used furanodiene which is a terpenoid isolated from *Rhizoma curcumae*, for their anticancer effects in zebrafish models. They observed that furanodiene showed anticancer effects in a pancreatic cell line (JF 305) and human breast cancer cells (MCF-7) transplanted into zebrafish. Furanodiene showed effective results through ROS production, anti-angiogenesis, apoptosis induction, and DNA strand breaks. 

Similarly, the artemisinin type compound can have anticancer activities against different types of tumors including leukemia, carcinomas of breast, kidneys, lungs, and ovaries, lymphoma, melanoma, and brain tumors [23,173,174]. Currently, reports of in vivo activities of *A. annua* are accumulating. One study reported anticancer activities of *A. annua* against four animal models aged 10 including a male cat with malignant fibrosarcoma, a male dog with malignant mesenchymal neoplasia, a female dog with breast cancer and another male dog with a malignant fibrosarcoma. The animals were treated with various doses of 150 mg/day (3 capsules), 450 mg/day (2 capsules), 450 mg/day (3 capsules), and 450 mg/day (2 capsules). All the animals showed complete reduction with no tumor relapse [175].

## 5. Regulatory Aspects of Herbal Anticancer Drugs

It is generally established that the drugs including the anticancer compounds require phase III clinical research trials for marketing permissions. The Food and Drug Administration (FDA) and European Medicines Agency (EMA) guidelines require at least one controlled trial in Phase III with statistically significant results for the green signal to market them [176]. Except for exceptional circumstances, all the drugs need to go through all the phases of trials according to the guidelines of international agencies such as the FDA and EMA. However, it has been observed that pharmaceutical companies deviate from the standard protocol and start testing new compounds on human subjects earlier than the defined timeline. The reason for such practices is to accelerate the approval of these compounds under the pressure of investors [176]. This means that the drug is presented for approval with insufficient data on its quality, safety, and efficacy. 

Although plant-based compounds have shown be less toxic compared to conventional synthetic compounds, there is growing evidence on the side effects of the unregulated use of these plants against different diseases. The problem is that there is insufficient data available regarding the quality, safety, and efficacy of herbal drugs. *F. indica*, for instance, has shown potent activity against breast cancer when tested in the MDA-MB-231 cell line [44]. *F. indica* is used traditionally to treat many disorders and people have even started the use of its herbal tea against breast cancer. However, the question remains that there are only a few reports available on the anticancer activity of the plant. Globally, the process of oncology drug development and marketing is regulated through the involvement of experts and an advisory process mediated by regulatory authorities [177].

There are several regulatory framework models available for prescribing such drugs but there is a need for harmony among regulating agencies and improvement in the regulation process. For instance, the FDA has recently adopted the questions and answers guidelines of the International Council for Harmonization on the nonclinical evaluation of drugs intended to treat cancer. These guidelines include 41 questions and answers which provide additional information about anticancer drug development and are aimed at bringing harmonization in the process of anticancer drug development [178]. It is, however, suggested that regulatory authorities, while bringing harmony with other agencies working for regulating anticancer herbal compounds, should increase the focus on combining information from traditional knowledge about that drug and the scientific studies on it [179].

Moreover, it is evident that plants of the same species grown in different areas vary in their profile of medicinal compounds [180]. This calls for the need to focus on the production of uniform and high-quality plants with a uniform metabolite profile that once tested is declared safe or unsafe once and for all. This might be achieved through the help of in vitro growth and biotechnological and genetic studies on these anticancer plants [181,182].

## 6. Modern Trends in Traditional Medicine Informatics and Opportunities for Anticancer Plant Products

With the advancement of information technology and bioinformatics, there is an increasing trend to build resources and databases that report herbal formulations, active components of the herb, and related information. There are several efforts like Chinese Medicine Integrated Database (TCMID) [183], Collective Molecular Activities of Useful Plants (CMAUP) [184], SymMap [185], encyclopedia of traditional Chinese medicine (ETCM) [186] etc. In addition, several researchers have developed strategies for in silico pharmacokinetic properties of molecules/drugs [187,188,189,190,191]. Such approaches are also applicable to phytochemicals and plant-based active drug components for their virtual screening, possible mode of action, and advanced drug discovery [192,193,194,195]. Several plant-based anticancer compounds have been evaluated using in silico and systems pharmacology tools [196,197,198,199,200,201]. The current study encourages further studies on anticancer active ingredients (of plant origin) for their in silico screening and pharmacokinetic activities. Considering the fact that plant-based drug formulations usually consists of several phytochemicals or even more than one plants. The major challenge on this direction would be to predict the role of phytochemicals other than active compounds and are present in the traditional medicine.

## 7. Conclusions

This detailed analysis of different plants showed that medicinal herbs promise a huge anticancer potential. This article comprehensively highlights the mechanism of antitumor action of some of the important plants. This is generally done through regulating signaling pathways. Many studies have reported inhibition of enzymes that stops tumor growth. These studies are mainly performed in human cell lines. It is highlighted that these plants play an important anticancer role through their different classes of secondary metabolites (Table 1). However, the study of these plants should not limit the study of a plethora of anticancer plants some of which are still unexplored. Studies are needed to highlight the mechanism of anticancer action of many already explored and many unexplored plants.

## Figures and Tables

**Table 1:** Some of the important anticancer medicinal plants, their active components, and in vitro and in vivo activity.

S.No.	Plant Name	Common Name	Parts Used	Extract Used (Aqueous/Methanolic etc.)	Active Components Used	Dose Concentration	Cancer Cell Line Applied To	Animal Models Applied To	References
1	*Allium sativum*	Garlic	Leaves	Aqueous extracts	Allicin, flavonoids, and phenolic components	20 mg/kg/0.2 mL	Wehi-164 tumor cells	Balb/c mice	[202]
2	*Alpinia galangal*	Lengkuas, greater galangal, and blue ginger	Rhizomes	Ethyl acetate extract	Chrysin	1.3 mg/kg	Murine daltons lymphoma ascite (dla) and human lung cancer (a549) cells	Balb/c mice	[203]
3	*Alstonia scholaris*	Blackboard or devil’s tree	Stem bark	Ethyl Alcohol extract	--	210 mg/kg	Hela cells lines	Swiss Albino mice	[204]
4	*Andrographis paniculata*	Creat or green chireta	Aerial parts	Methanolic extract	Diterpenes	10 µg/mL	Cancer cell lines sw620 and a498	Swiss Albino mice	[205]
5	*Angelica archangelica*	Garden angelica, wild celery, and Norwegian angelica	Root and rhizome	Ethanolic extract	Angelicin	500mg/kg	Mcf7 and 4t1 cell lines	Female balb/c mice	[206]
6	*Aralia elata*	Chinese angelica-tree, Japanese angelica-tree, and Korean angelica-tree	Leaves	Ethanol extract	*--*	300 mg/kg	Mcf-7 cells	Tumor bearing-nude mice	[207]
7	*Artemisia annua*	Sweet wormwood, sweet annie, and sweet sagewort	*--*	*--*	Artemisinin	0.02%	Breast cancer	Rats	[168]
8	*Asclepia scurassavica*	Tropical milkweed	Shade dried leaves	Ethyl acetate and methanolextract	Β-sitosterol	10–20 mg/kg b.w.	Human colo 320 dm and monkey vero cell lines	Male wistar rats	[208]
9	*Astragalus membranaceus*	Mongolian milkvetch	*--*	*--*	Polysaccharide	400 mg/kg	Liver cancer	H22 hepatocarcinoma transplanted balb/c mice	[209]
10	*Copaifera multijuga*	Hayne oil, Copaiba	Trunk of the tree	Oil resin	Clerodane Diterpenes	2 g/Kg	B16f10 melanoma cells	Male C57/black mice	[210]
11	*Coptidis rhizoma*	Huanglian, Copaiba, and Copaibera	*--*	*--*	Berberine	200 µM and 400 µM	Human hepatic carcinoma cell lines HepG2 and mhcc97-l.	*--*	[211]
12	*Curcuma longa*	Turmeric	--	--	Curcumin	75 µM	Ht-29 colon cancer cells of human	*--*	[32]
13	*Elephantopus scaber*	Elephant′s Foot	*--*	Dimethyl sulfoxide extract	Deoxyelephantopin (doe)	25mg/kg	Murine ehrlich ascites carcinoma (eac)	Male swiss albino mice	[212]
14	*Fagonia schweinfurthii*	bush candle	Whole plant	Ethanolic extract	Carbon tetrachloride (ccl4)	200 µg/mL	HepG2 cell line	Male albino rats	[213]
15	*Garcinia indica*	Kokum	Fruits	Ethanol extract	Garcinol	<1 μM	Ht-29 and hct-116 colon cancer cells	*--*	[50]
16	*Garcinia oblongifolia*	Lingnan garcinia	Branch	Methanol extract	Xanthone	1000 μg/mL	Mcf-7 breast cancer cell line	*--*	[49]
17	*Garcinia preussii*	--	Fruits and leaves	Meohextract	Benzophenones		Du145, hela, ht-29, and a431 cell lines	*--*	[214]
18	*Hedyotis diffusa*	Snake-needle grass	*--*	*--*			Hela cells	Nude mice xenograft	[215]
19	*Hedyotis* spp.	*--*	*Aerial parts, stem and leaves*	Methanol extract	*--*	20 μM	Cem-ss cell line	*--*	[54]
20	*Kaempferia parviflora*	Black ginger	Rhizomes	Ethanolic extract	*--*	1 mg/mL	Ovarian cancer cell line, skov3	*--*	[216]
21	*Litchi chinensis*	litchi or lychee	Fruit pericarp	Ethanolic extract	Polyphenolic compounds	0.3 mg/mL	Human smmc-7721 hepatocellular carcinoma cell Line	Murine hepatoma bearing-mice	[217]
22	*Menyanthes trifoliata*	Bogbean, Buckbean, and Marsh Trefoil	Aerial part and root	Aqueous methanol extract	Polyphenolic compounds	1.5 mg/mL	Grade iv glioma cells	*--*	[218]
23	*Morus alba*	white mulberry	Root	N-hexane and methanolextracts.	Albanol a	30 µM	HL-60 (human leukemia) and Crl1579 (human melanoma) cell lines	*--*	[69]
24	*Morus nigra*	Black mulberry or blackberry	Aerial parts	dimethyl sulfoxide extract	Phenolic compounds especially Ascorbic acid and chlorogenic acid	1000 μg/mL	human prostate adenocarcinoma (PC-3)	*--*	[219]
25	*Nitraria retusa*	Salt tree or Nitre bush	Leaves	Chloroform extract	Β-sitosterol and palmitic acid	50 mg/Kg b.w	B16-f10 cells lines	Balb/c mice	[220]
26	*Paeonia lactiflora*	Chinese Peony	Root	Aqueous extract	--	15 mg/mL	Human hepatoma cell lines (HepG2 and hep3b)	*--*	[221]
27	*Paris polyphylla*	Herb Paris	Rhizomes	Methanol extract	Steroidal saponins	7.5 mg/kg	A549 cell line	Tumor-bearing c57bl/6 mice	[163]
28	*Perilla frutescens*	Beafsteak plant	Leaves	Meoh extract	Isoegomaketone	10nmol/l	Huh-7 hepatoma cell carcinoma	Tumor-xenograft nude mice	[164]
29	*Perilla frutescens*	Beafsteak plant	Leaf	*--*	Rosmarinic acid	105 µg/mL	Human hepatoma (HepG2) cells	*--*	[89]
30	*Platycodon grandiflorus*	balloon-flower	Root	Platycodin d was dissolved in Phosphate-buffered saline	Platycodin D	8 µg/mL	Human breast cancer cell line, mcf-7	*--*	[98]
31	*Pleurotus pulmonarius*	Indian Oyster, Italian Oyster, Phoenix Mushroom, or the Lung Oyster	Edible part	Aqueous extract	*--*	20 mg/kg	Huh7 liver cancer cells	Nude mice	[222]
32	*Rabdosia rubescens*	Bing Ling Cao, BlushredRabdosia, and Isodonrubescens	*--*	*--*	Oridonin	30 μmol/L	Human gallbladder cancer cell lines sgc996 and noz	Athymic nude mice	[113]
33	*Rhodamnia rubescens*	Scrub stringybark, brush turpentine, or brown malletwood	*--*	*--*	Tetracycline diterpenoidoridonin	50 μM	Human breast (mcf-7 and mda-mb-231) cancer cells	*--*	[115]
34	*Scutellaria barbata*	Barbed Skullcap	*--*	*--*	Polysaccharides	40 µg/mL	95-d cell line	Xenograft model	[162]
35	*Scutellaria baicalensis*	Baikal skullcap	Root	Aqueous extract	Baicalin	100 µg/mL	Human oral squamous cell carcinoma (oscc) cell line	*--*	[127]
36	*Tripterygium wilfordii*	Thunder god vine	*--*	*--*	Triptolide	250 nmol/L	Neuroblastoma cell lines (n2a and sknsh)	Neuroblastoma (nude mice model)	[145]
37	*Tussilago farfara*	Coltsfoot	Flower buds	Methanol extract	Quercetin-glycosides		Ht-29 human colon cancer cells	*--*	[149]
38	*Wedelia chinensis*	Chinese Wedelia	Leaves	Essential oils	Carvocrol and trans-caryophyllene		B16f-10 melanoma metastatic cell line	C57bl/6 mice	[153]
39	*Zuojin wan*		*--*	Aqueous extract	Palmatine, berberine, epiberberine, and coptisine	10 mg/mL	S180 tumor cells	Chinese kunming (km) mice	[223]
